# Thermosensitive hydrogel‐based GPR124 delivery strategy for rebuilding blood‐spinal cord barrier

**DOI:** 10.1002/btm2.10561

**Published:** 2023-06-06

**Authors:** Jiawei Shu, Chenggui Wang, Yiqing Tao, Shaoke Wang, Feng Cheng, Yuang Zhang, Kesi Shi, Kaishun Xia, Ronghao Wang, Jingkai Wang, Chao Yu, Jiangjie Chen, Xianpeng Huang, Haibin Xu, Xiaopeng Zhou, Haobo Wu, Chengzhen Liang, Qixin Chen, Shigui Yan, Fangcai Li

**Affiliations:** ^1^ International Institutes of Medicine The Fourth Affiliated Hospital, Zhejiang University School of Medicine Yiwu Zhejiang People's Republic of China; ^2^ Department of Orthopedics Surgery The Second Affiliated Hospital, School of Medicine, Zhejiang University Hangzhou Zhejiang People's Republic of China; ^3^ Orthopedics Research Institute of Zhejiang University, Zhejiang University Hangzhou Zhejiang People's Republic of China; ^4^ Key Laboratory of Motor System Disease Research and Precision Therapy of Zhejiang Province Hangzhou Zhejiang People's Republic of China; ^5^ The Second Affiliated Hospital and Yuying Children's Hospital of Wenzhou Medical University Wenzhou Zhejiang People's Republic of China

**Keywords:** blood‐spinal cord barrier, controlled release, energy metabolism, GPR124, spinal cord injury, thermosensitive hydrogel

## Abstract

Spinal cord injury (SCI) causes blood‐spinal cord barrier (BSCB) disruption, leading to secondary damage, such as hemorrhagic infiltration, inflammatory response, and neuronal cell death. It is of great significance to rebuild the BSCB at the early stage of SCI to alleviate the secondary injury for better prognosis. Yet, current research involved in the reconstruction of BSCB is insufficient. Accordingly, we provide a thermosensitive hydrogel‐based G protein‐coupled receptor 124 (GPR124) delivery strategy for rebuilding BSCB. Herein, we firstly found that the expression of GPR124 decreased post‐SCI and demonstrated that treatment with recombinant GPR124 could partially alleviate the disruption of BSCB post‐SCI by restoring tight junctions (TJs) and promoting migration and tube formation of endothelial cells. Interestingly, GPR124 could also boost the energy metabolism of endothelial cells. However, the absence of physicochemical stability restricted the wide usage of GPR124. Hence, we fabricated a thermosensitive heparin‐poloxamer (HP) hydrogel that demonstrated sustained GPR124 production and maintained the bioactivity of GPR124 (HP@124) for rebuilding the BSCB and eventually enhancing functional motor recovery post‐SCI. HP@124 hydrogel can encapsulate GPR124 at the lesion site by injection, providing prolonged release, preserving wounded tissues, and filling injured tissue cavities. Consequently, it induces synergistically efficient integrated regulation by blocking BSCB rupture, decreasing fibrotic scar formation, minimizing inflammatory response, boosting remyelination, and regenerating axons. Mechanistically, giving GPR124 activates energy metabolism via elevating the expression of phosphoenolpyruvate carboxykinase 2 (PCK2), and eventually restores the poor state of endothelial cells. This research demonstrated that early intervention by combining GPR124 with bioactive multifunctional hydrogel may have tremendous promise for restoring locomotor recovery in patients with central nervous system disorders, in addition to a translational approach for the medical therapy of SCI.

## 1 INTRODUCTION

Spinal cord injury (SCI) has two pathophysiological phases: primary and secondary phases. In the primary phase, structural disturbance, especially blood‐spinal cord barrier (BSCB) disrupted caused by the trauma, which is one of the most crucial triggers of secondary phase.^1^, ^2^ The permeability of BSCB increased due to the disruption of BSCB post‐SCI followed by peripheral inflammatory cells and molecules crossing into the lesion, contributing to the subsequent damage, and further aggravating the BSCB disruption.^3^ Accordingly, reconstructing the BSCB at the early stage of SCI may provide a more suitable microenvironment for long‐term nerve repair. However, the major clinical treatments and interventions for SCI rarely involve the BSCB reconstruction due to the elusive therapeutic and observational time window, post‐traumatic vessel regression, and nonfunctional neovascularization.^4^, ^5^

BSCB comprises nonfenestrated endothelial cells (ECs), pericytes, astrocytic end‐feet processes, and the basement membrane, which restricts paracellular and transcellular trafficking in the central nervous system. The BSCB function is similar to the blood–brain barrier in protecting the brain from toxins and infections and stimulating neuronal function. Yet, BSCB has unique structural and functional features such as glycogen deposits, different permeability, and protein expressions, which give it a unique vulnerability to pathological insults.^5^

G protein‐coupled receptor 124 (GPR124) is an orphan G‐protein coupled receptor (GPCR),^6^ which is absolutely needed for sprout and movement of blood vessels into neuroepithelium during the integrity of blood–brain barrier.^7^ Interestingly, GPR124 is also a potential target for blood–brain barrier devastation in stroke and glioblastoma,^8^ but whether the BSCB disruption is related to GPR124 and whether it can rescue SCI remains unknown. To the best of knowledge, the unique function of GPR124 also plays a similar role in developing vasculature.^9^ Therefore, we have speculated that GPR124 might be able to rebuild the BSCB via angiogenesis post‐SCI. Although the BSCB of the lesion is disrupted post‐SCI, neither subcutaneous nor intravenous injection of GPR124 is effective for GPR124 is a macromolecular protein and lacks the capability to penetrate the BSCB normal site. Accordingly, in situ delivery could facilitate GPR124 evade the BSCB. However, the effect is predictably confined due to the short half‐life, loss of in situ blood circulation, and matrix metalloproteinase (MMP) processing.^10^

Considering that GPR124 could be combined with heparin,^10^ and heparin could also preserve growth factors with high‐affinity heparin‐bound from degradation. In addition, laponite/heparin hydrogel could load more growth factors and release it slowly and sustainably.^11^, ^12^ Protecting GPR124 with heparin seems to be a good idea, for the combination of GPR124 and heparin may also reduce the degradation of GPR124 and prolong its potential effect. However, the simple combination was still unable to change its water‐solubility to fully meet our requirements of the ability of sustained release in situ.

In recent years, biomaterials‐based drug/cell delivery systems for restoring SCI have aroused wild interest, which is due to the unique space structures, easily modified characteristics and multifunctional properties of biomaterials, such as nanoparticles, liposomes, micelles as well as hydrogels.^13^, ^14^, ^15^, ^16^ Therefore, applying hydrogels in SCI treatment has various advantages: (1) hydrogels are easy to be modified to meet the challenges of the complex microenvironment in vivo^17^; (2) the three‐dimensional network structure of hydrogels could be used for the long‐time controlled release of drugs^18^; (3) hydrogels could be used as scaffolds to guide the growth of cells and axons.^19^ Consequently, in situ delivery by using hydrogels could easily load biomacromolecules, expanding their duration as well as maintaining their activity.

Poloxamer, as one of the classic hydrogels, has the mainly characteristics of hydrogels, and also owns unique merits: First, it could covalently bind to heparin through several chemical modification steps to form a hydrogel with three‐dimensional networks. Second, it is certified by the Food and Drug Administration (FDA), which may speed clinical translation. Next, the excellent effect on temperature sensitivity according to the concentration makes it more suitable for filling injured tissue cavities in situ, resulting in the protection of lesion site. Last, but not least, it has the ability of sustained release and injectability. Accordingly, we enabled poloxamer (P) as the host of our biomaterial. Heparin is used as a bridge between GPR124 and poloxamer. Taken together, an injectable and thermosensitive heparin‐poloxamer (HP) hydrogel was prepared here to load GPR124 (HP@124), which protects GPR124 from degradation and delivery in a sustained manner.

Herein, we demonstrated the role of GPR124 on BSCB reconstruction post‐SCI, together with the synergistic effect of HP hydrogels, taking it further in vivo and in vitro. Moreover, we also found that adding GPR124 could attenuate the tight junctions (TJs) loss of endothelial cells, together with boosting migration and tube formation by restoring energy metabolism. Meanwhile, various comprehensive evaluations on function, morphology, and histology were carried out to assess the long‐term effect of the HP@124 hydrogel. In general, a thermosensitive hydrogel‐based GPR124 delivery strategy for early intervention of SCI was designed with notable physicochemical effects, which inhibits the disruption of BSCB and promotes motor function post‐SCI.

## 2 METHODS

### 2.1 HP@124 hydrogel preparation

First, we used 1‐ethyl‐3‐(3‐dimethylaminopropyl) carbodiimide (EDC) (Sigma‐Aldrich, USA) and N‐hydroxylsuccinimide (NHS) (Sigma‐Aldrich, USA) approach to synthesize HP. In brief, a mono amine‐terminated P (MATP) was prepared through the reaction between Poloxamer 407 (Sigma‐Aldrich, USA) and 4‐nitrophenyl chloroformate (Aladdin, China) under the condition of diamino ethylene (Sigma‐Aldrich, USA). Next, the reaction of heparin (Solarbio, China) and MATP in 2‐(N‐morpholine) sulphonic acid (Aladdin, China) was performed by the coupling of EDC and NHS at room temperature for 24 h. After dialysis and lyophilization, the HP powder was obtained. Next, the HP hydrogel solution (1.6 × 10^3^ mg/mL) was obtained by dissolving the lyophilized powder in fresh saline (4°C), and then GPR124 solution (500 μg/mL; R&D systems, Inc, Minneapolis, USA) was added under the aseptic conditions. The final concentration of GPR124 and HP was 50 μg/mL and 160 mg/mL respectively.

### 2.2 HP@124 hydrogel characterization

The HP hydrogel's structure was characterized by Fourier transform infrared spectroscopy (FTIR) (NICOLET 6700, Thermo). And the thermo‐sensitivity was tested by observing the hydrogel state from 4°C to 37°C and back to 4°C. In addition, dynamic rheology was carried out on a rheometer (MCR 302; Anton Paar, Graz, Austria) by recording the storage and loss moduli, supported by a parallel plate (25 mm). The frequency was set to 10 rad/s; fixed strain was set to 1%.

### 2.3 Controlled release experiment

The P hydrogel and HP@124 hydrogel were prepared by adding with 1 μg GPR124, respectively. Then, these two hydrogels were added into tubes, respectively, followed with equal volume of normal saline and incubated at 37°C. The supernatant was accumulated and replaced by the equivalent volume of normal saline at specific time points. The total concentrations of released GPR124 were measured using a micro bicinchoninic acid (BCA) protein assay kit according to the manufacturer's instruction (Thermo Scientific, USA).

### 2.4 Animal surgery and drug intervention

One hundred and eight mature female SD rats (for facilitating urination management and eliminating gender differences in SCI repair) were prepared under regulated environmental conditions for the subsequent in vivo evaluations, which were purchased from the SLAC Laboratory Animal Company (Shanghai, China). The weight of rats was controlled from 220 to 240 g. All the animal procedures were approved by the Institutional Animal Care and Use Committee of Zhejiang University. Rats were anesthetized by intraperitoneal injection of 1% (w/v) pentobarbital sodium (4 mL kg^−1^) before the operation. A T9 laminectomy was implemented subsequently to fully expose the spinal cord, followed with a moderate crushing injury of spinal cord performed by a vascular clip for 1.5 min (30 g forces, Oscar, China).^20^ After injury, orthotopic injection of 10 μL different contents (normal saline, HP hydrogel, a free 500 ng GPR124 solution or HP hydrogel loaded with 500 ng GPR124) was performed to cover the lesion site of the corresponding groups of rats by a micro syringe (26‐gauge) (Hamilton, Switzerland). As for the sham group, only laminectomy was operated. A 4 × 10^6^ unit dose of penicillin solution was injected intraperitoneally in each animal within 1‐week post‐surgery. Bladder emptying was also undertaken twice a day.

### 2.5 Tissue preparation

Animals were anesthetized and the spinal cord was harvested at 1, 2, 3, 7, and 28 days after treatment. For paraffin sections (5 μm thick, pre‐sectioning to the vicinity of the injured center), 1 cm segment of the harvested spinal cord including lesion site was anatomized, followed by fixation with 4% paraformaldehyde (PFA) overnight. Paraffin embedment was performed subsequently. For the western blot (WB) experiment, 0.5 cm segment of the spinal cord including lesion epicenter was dissected and preserved at −80°C after harvested.

### 2.6 Evaluation of BSCB permeability

The BSCB permeability was assessed by Evans Blue (EB) staining assay according to previous reports.^12^ At 3 days post‐SCI, 0.15 mL of 2% EB (Sigma, St. Louis, MO) in normal saline was injected into the rats via intravenous injection. One hour post injection, intra‐cardiac perfusion with normal saline was performed to sacrifice the anesthetized rats. The spinal cord was harvested for subsequent observation after adequate perfusion with saline. The EB content was quantified by soaking the harvested spinal cord into methanide for 1 day at 60°C and measured as absorbance of supernatant at 632 nm.

### 2.7 Histology and immunofluorescence

Paraffin sections were arranged as described above. Hematoxylin and eosin (H&E) staining (Beyotime Biotech Inc, Shanghai, China) of sections was performed for the evaluation of histopathology. Immunofluorescence test was implemented using primary antibodies targeting the corresponding proteins at 4°C overnight. After adequately washed by phosphate‐buffered saline with 0.05% Tween‐20 (PBST), the sections were incubated with secondary antibodies for 1 h at 37°C. Next, phosphate‐buffered saline (PBS) (Biosharp, China) was used to rinse the sections for three times, followed with 4′, 6‐diamidino‐2‐phenylindole (Sigma‐Aldrich, USA) for 6 min. At last, the sections were sealed with a coverslip after washed with PBS. All primary and secondary antibodies used here are listed in Table S1.

### 2.8 Functional behavior assessment

Basso–Beattie–Bresnahan (BBB) locomotion scale was carried out to assess the recovery of motor function, of which the points range from 0 to 21 depending on the extent and coordination of hindlimb joints' movements.^21^ Meanwhile, footprint test was also carried out to evaluate the continuous walking behavior of rats. In brief, rats were printed by blue (posterior limb) or red (fore limb) inks^22^ and were motivated to pass through a narrow pipe which could not turn back.

### 2.9 Cell culture and drug intervention

Human cerebral microvascular endothelial cell line (HCMEC/D3) and human umbilical vein endothelial cell (HUVEC) (iCell, Shanghai, China) were cultured in high glucose Dulbecco modified Eagle medium (DMEM) medium (Sigma, USA) with 10% fetal bovine serum (Gibco, USA). For further detection of the effect of GPR124 and HP@124 hydrogel, we divided cells into four groups: sham group (DMEM), tert‐butyl hydroperoxide (TBHP) group (add with 100 μM TBHP (Sigma‐Aldrich, USA)),TBHP + GPR124 group (add with 100 μM TBHP and 50 μg/mL GPR124), TBHP + HP@124 group (using a transwell chamber with 8 μm filter inserts, the upper chamber was filled with 500 μL HP@124, along with the lower chamber seeded with cells and 100 μM TBHP).

#### 2.9.1 Migration assay

Vertical migration test of HCMEC/D3s was performed by using transwell chamber with 8 μm filter inserts (Corning, USA).^23^ Cells were digested and diluted to the concentration of 2 × 10^5^ cells/mL after pretreatment, and then seeded into the superior chamber with high glucose DMEM medium only, whereas 600 μL high‐glucose DMEM medium containing 10% fetal bovine serum was added into the inferior chamber. The cells on the inferior surface were fixed by 4% formaldehyde, followed with staining of 0.1% crystal violet (Beyotime Biotech Inc., Shanghai, China).

#### 2.9.2 Wound migration assay

Pretreated HCMEC/D3s were seeded into a six‐well plate until grown into a confluence ratio of over 95%. Afterward a straight shape was scratched by a 200 μL tip. After observation and record, cells were rinsed with PBS and replaced with equal volume of the medium.^23^ Images were captured again for comparison per 12 h.

#### 2.9.3 Tube formation assay

Pretreated HUVECs were seeded in a μ‐Slide Angiogenesis (ibidi, Germany) pre‐coated with 10 μL Matrigel (Corning, USA) per well and incubated at 37 °C. After 12 h, the vascular network was recorded by brightfield microscope for following quantitative analysis.

#### 2.9.4 Permeability assay

The permeability experiment of HCMEC/D3s was performed using a transwell chamber with 0.4 μm filter inserts (Corning, USA). Here, fluorescein isothiocyanate (FITC)‐dextran (4 kDa, Sigma, USA) was employed to examine the penetration of monolayer barrier of HCMEC/D3s. Pretreated cells were filled in the superior chamber to reach the confluence ratio of 100%, subsequently, adding with 100 μL FITC‐dextran solution (1 mg mL^−1^) in the superior chamber, meanwhile, 500 μL high glucose DMEM medium was filled in the inferior chamber. At last, collected the medium in the inferior chamber after 2 h and evaluated by fluorescence.^24^

#### 2.9.5 RNA sequencing analysis

HCMEC/D3s were treated with different media (TBHP, TBHP + GPR124, and TBHP + HP@124 as mentioned before) for 3 days. Next, lysed by Trizol (Takara Bio Inc, Japan), total RNA extraction was collected for the following RNA sequencing analysis (Metware Biotechnology Co., Ltd., Wuhan, China). In order to elucidate the role of genes modulated by the gel, gene ontology (GO) and Kyoto Encyclopedia of Genes and Genomes (KEGG) analysis were carried out.

#### 2.9.6 Seahorse test

Pretreated HCMEC/D3s were seeded in Seahorse XF96 plates (Agilent, China) with the amount of 20,000 cells per well. Cells were incubated with nonglucose medium for 1 h before undergoing the glycolysis stress evaluation (Seahorse XF96 analyzers, Agilent, China) by detecting the extracellular acidification rate (ECAR) of cells. Detection was carried out per 5 min before and after sequentially adding with glucose, oligomycin and 2‐dexoy‐d‐glucose (2‐DG) (Agilent, China). Next, cells were incubated with normal medium for 1 h before the mitochondrial stress evaluation by detecting the oxygen consumption rate (OCR) of cells. And detection was carried out per 5 min before and after sequentially adding with oligomycin, carbonyl cyanide 4‐(trifluoromethoxy)phenylhydrazone (FCCP) and rotenone/antimycin A (Agilent, China). Wave software and Graphpad were employed to process and analyze the data obtained.^25^

### 2.10 WB analysis

Protein extracts were obtained by cells and spinal cord tissues mentioned before. After lysis by radioimmunoprecipitation assay (RIPA) lysis buffer (Beyotime Biotech Inc., Shanghai, China) that contains 1% phenylmethanesulfonyl fluoride (PMSF) (Beyotime Biotech Inc., Shanghai, China), the solutions were centrifuged at 12,000*g* for 15 min at 4°C. The supernatant was needed as subsequent experimental protein samples, of which concentration was examined by BCA protein assay kit (Beyotime Biotech Inc., Shanghai, China). After separated by 10% sodium dodecyl sulfate‐polyacrylamide gel electrophoresis (SDS‐PAGE), polyvinylidene difluoride membranes (Millipore, MA) were used to receive the electro‐transferred protein samples. Subsequently, the membranes were blocked by 10% nonfat milk dissolved in PBST for 1 h. Then, the membranes were incubated with the corresponding primary antibodies at 4°C overnight. After washing three times by tris buffered saline with 0.05% tween‐20 (TBST), the samples were incubated with horseradish peroxidase‐conjugated specific antibodies for 60 min at room temperature. Immunoreactive bands were visualized by Chemi DocXRS + Imaging System (Bio‐Rad), and ImageJ software was applied to calculate gray levels. All experiments were performed in triplicate. All primary and secondary antibodies used here are listed in Table S2.

### 2.11 Transmission electron microscopy

The animals were anesthetized by intraperitoneal injection of 1% sodium pentobarbital (4 mL kg^−1^) and transcardially perfused with normal saline, followed by 4% PFA and 2% glutaraldehyde in 0.1 M PBS. The samples containing the lesion site of the spinal cord were harvested and post‐fixed with 2.5% glutaraldehyde in 0.1 M PBS before fixation with 1% osmium tetroxide. After rinsing with sterilized double distilled water, the samples were dehydrated with a graded series of ethanol and then embedded in pure epoxy. Ultrathin sections (70–90 nm) were obtained with an ultramicrotome (EMUC7, Leica, Germany) and doubly stained with lead citrate and uranyl acetate before observation under a transmission electron microscopy (TEM) (EM 10C, Zeiss, Germany).

### 2.12 Statistical analyses

All the data are shown as the mean ± SD. All data was subject to outlier (ROUT method) and normality (Shapiro–Wilk) testing. Parametric, normal datasets were analyzed using the Student's *t*‐test and analysis of variance (ANOVA) test with post hoc Tukey's testing. The significance threshold was set at a *p value < 0.05 and **p < 0.01. Values with different superscripts are significantly different (*p* < 0.05).

## 3 RESULTS

### 3.1 Blood‐spinal cord barrier breakdown along with the expression of GPR124 decreased and tight junctions disruption post‐SCI

**FIGURE 1:**
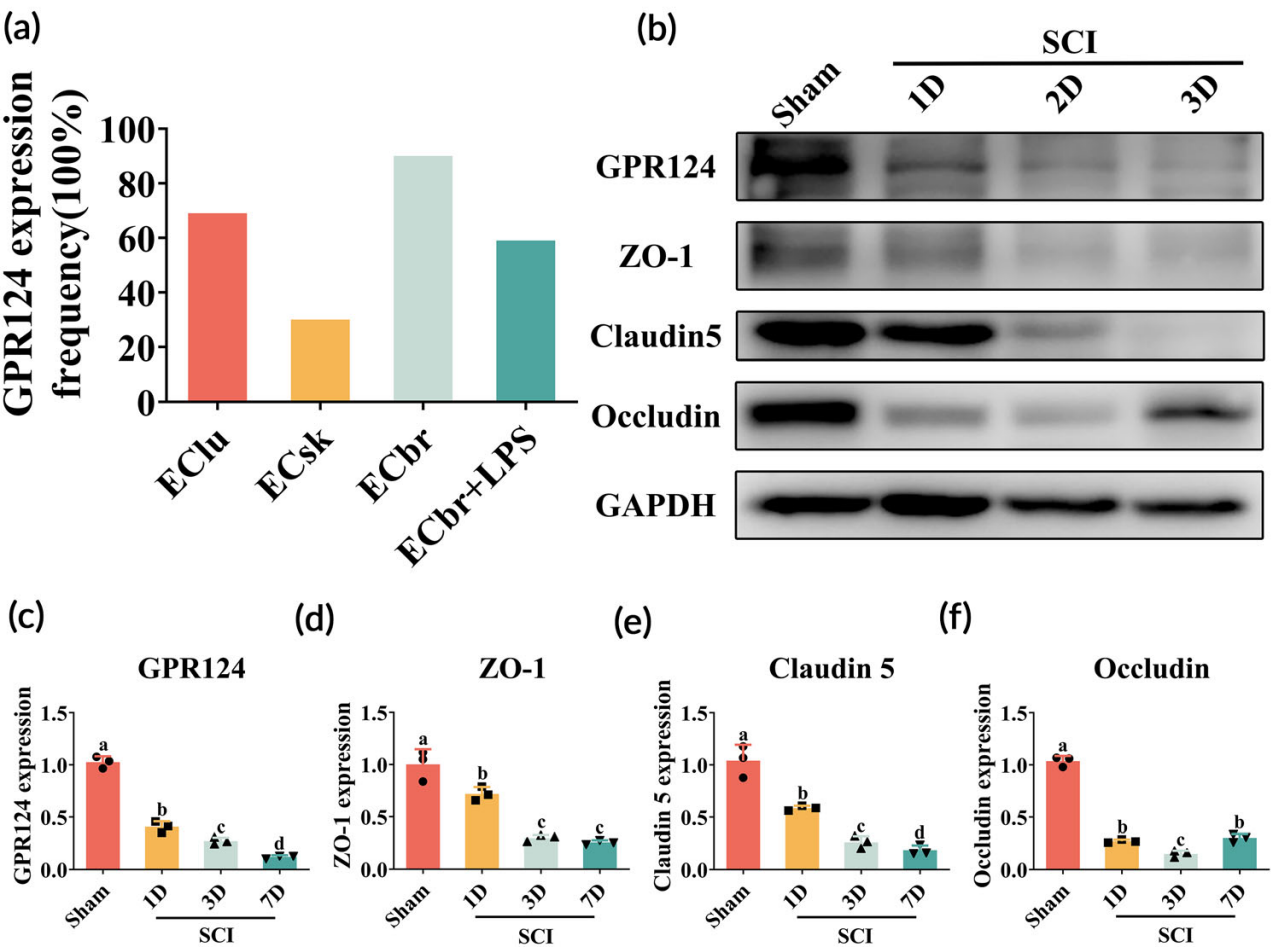
The relationship between decreased expression of G protein‐coupled receptor 124 (GPR124) in endothelial cells (ECs) and tight junction proteins post‐spinal cord injury (SCI). (a) The expression frequency in % of GPR124 in EClu (ECs in lung), ECsk (ECs in skeletal muscle) and ECbr (ECs in brain) from normal mice and lipopolysaccharide (LPS)‐treated mice. (b–f) Western blot analysis of GPR124 and tight junction proteins at 1, 2, and 3 days post‐SCI and the related quantification results, values with different superscripts are significantly different (*p* < 0.05, one‐way analysis of variance (ANOVA) test, *n* = 3).

Differential expression profiles of GPCR in different types of endothelial cell (EC) were obtained from the research carried out by Kaur et al. and their uploaded database in the National Center for Biotechnology Information Gene Expression Omnibus database (GSE97955).^26^ ECs obtained from the brain showed higher expression of GPR124. In addition, in response to acute inflammatory activation, the expression of GPR124 was downregulated in ECs obtained from the brain (Figure 1a). Whether these patterns of GPR124 are similar to SCI remains unknown.

To indicate the potential role of GPR124 during SCI, we detected the alterations of GPR124 and TJ proteins in the spinal cord post‐SCI by western blot (WB) (Figure 1b). We found that the protein level of TJ, including occludin, claudin 5, and zonula occludens‐1 (ZO‐1), decreased significantly over time post‐SCI (Figure 1d–f). More importantly, the protein level of GPR124 is also downregulated by time post‐SCI (Figure 1c). Together, these results imply the potential relationship between GPR124 and BSCB disruption post‐SCI.

### 3.2 Fabrication and characterizations of heparin‐poloxamer and heparin‐poloxamer@124 hydrogel

**FIGURE 2:**
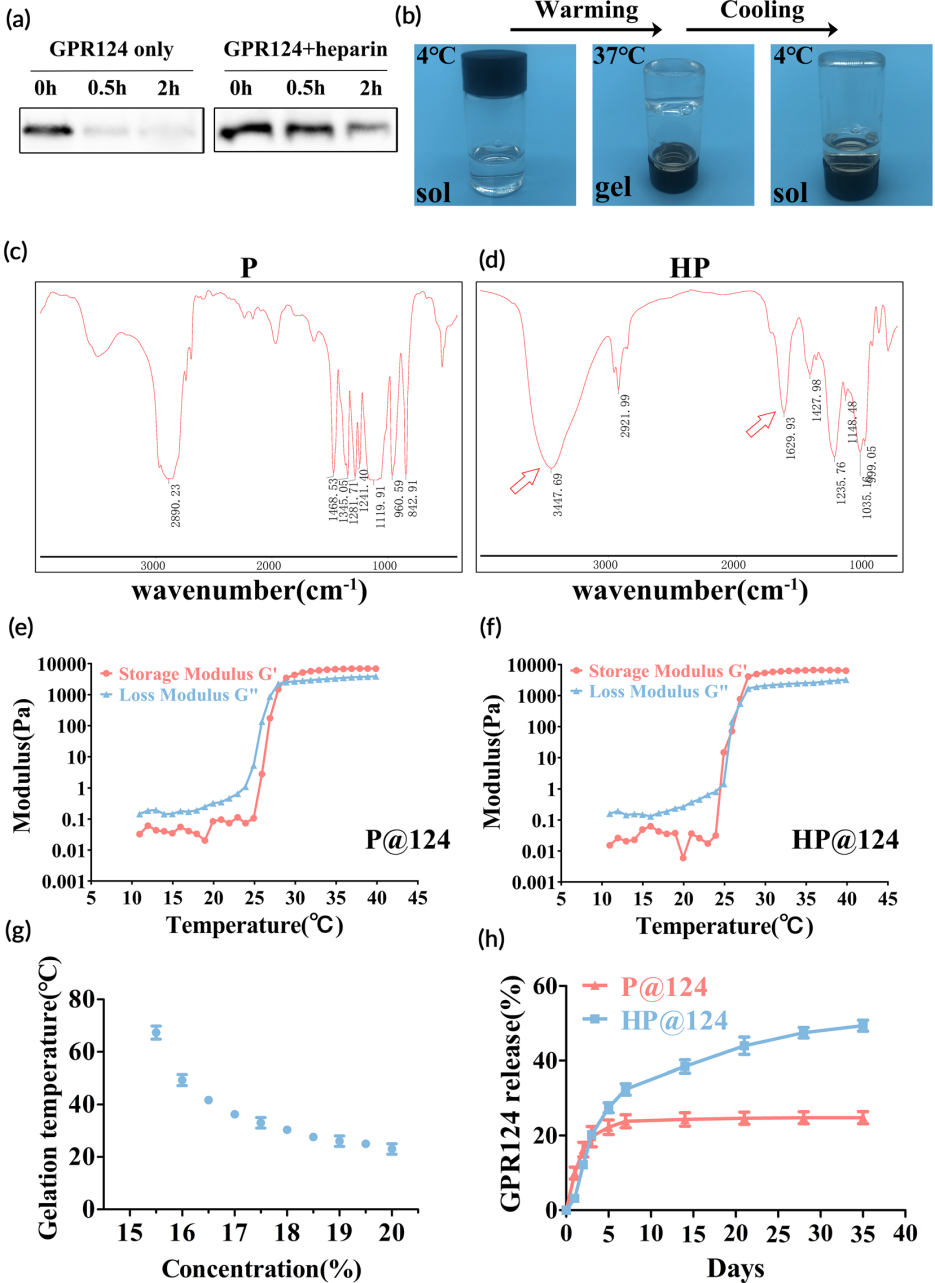
Heparin‐poloxamer (HP)@124 hydrogel characterizations. (a) The degradation rate of G protein‐coupled receptor 124 (GPR124) with or without heparin by Western blot (WB). (b) The sol–gel transformation of HP@124 hydrogel with temperature alterations. (c,d) Fourier transform infrared spectroscopy (FTIR) spectra of P and HP hydrogel. (e,f) The alteration of G′ and G″ moduli of P@124 and HP@124 hydrogels with temperature ranges from 10°C to 40°C. (g) The HP gelation temperature under different concentrations. (h) Cumulative release of GPR124 in two delivery strategies.

First, our experiments revealed that the protein of GPR124 degraded within 2 h in PBS (Figure 2a). Interestingly, researchers have shown the ability of GPR124 to interact with heparin.^10^ So next, we verified whether heparin could protect GPR124 from degradation by WB (Figure 2a). We have shown that the P could form an amide bond with heparin to obtain HP hydrogel in previous works.^12^ Eventually, we came up with the idea of combining them together.

To obtain HP hydrogels, we conjugated MATP to heparin by the coupling of EDC and NHS. Fourier transform infrared spectroscopy (FTIR) was employed to detect the chemical conformation of HP. As illustrated in FTIR spectra (Figure 2c,d), at about 1600 cm^−1^, a new absorption peak belonging to the carbonyl vibration of HP complex could be identified as one of the characteristics of HP. Meanwhile, compared with P, hydroxyl groups of conjugated heparin could also be observed at 3300–3600 cm^−1^. According to the spectral results, we successfully synthesized HP hydrogels by EDC/NHS method. Besides, the gelation temperature could be modulated by changing the concentration of HP (Figure 2g). In view of the body temperature of animals and humans, it is advocated that HP hydrogel with the concentration of 17 wt% as the ideal hydrogel for the subsequent experiments. As illustrated in Figures 2b and S1A,B at 4°C, HP was under a liquid (sol) condition and exhibited injectable characteristics; when heating to 37°C, it quickly transformed into the hydrogel (gel) condition. Concurrently, it transformed back to a solution (sol) condition once down to 4°C. We also investigated the gelation process of P loaded with GPR124 (P@124) and HP loaded with GPR124 (HP@124) by testing storage moduli (G′) and loss moduli (G″), which represent the alteration of viscosity and elasticity respectively (Figures 2e,f and S1C,D). Both delivery strategies indicated a moderate sol‐to‐gel transformation between 25°C and 30°C, enabling simple modulation and a wide range of applications. The similar intersection of amplitude sweep of ~9% revealed that both of them were comparatively soft and appropriate for biological demands on the spinal cord. Furthermore, we tested the in vitro release situation of GPR124 from HP or P hydrogels (Figure 2h). An initial burst release (~150 ng, 15%) of GPR124 from P@GPR124 appeared within 2 days, in comparison, a continuous release of GPR124 was carried out in HP@124 hydrogel. And in day 28, about 50% (500 ng) of the loaded GPR124 was released from the HP@124 hydrogel, which was 2 times greater than that in the P@GPR124 hydrogel. Subsequently, a sustained release behavior of GPR124 was observed in the HP hydrogel, achieving temporal and spatial regulation in the delivery strategy of GPR124.

Together, we demonstrated that the HP@124 hydrogel is thermosensitive and suitable for the spinal cord. Besides, containing heparin could immobilize and preserve GPR124 from degradation, which enables the release of GPR124 sustainably.

### 3.3 Role of heparin‐poloxamer@124 hydrogel on migration, angiogenesis, and energy metabolism of endothelial cells in vitro

**FIGURE 3:**
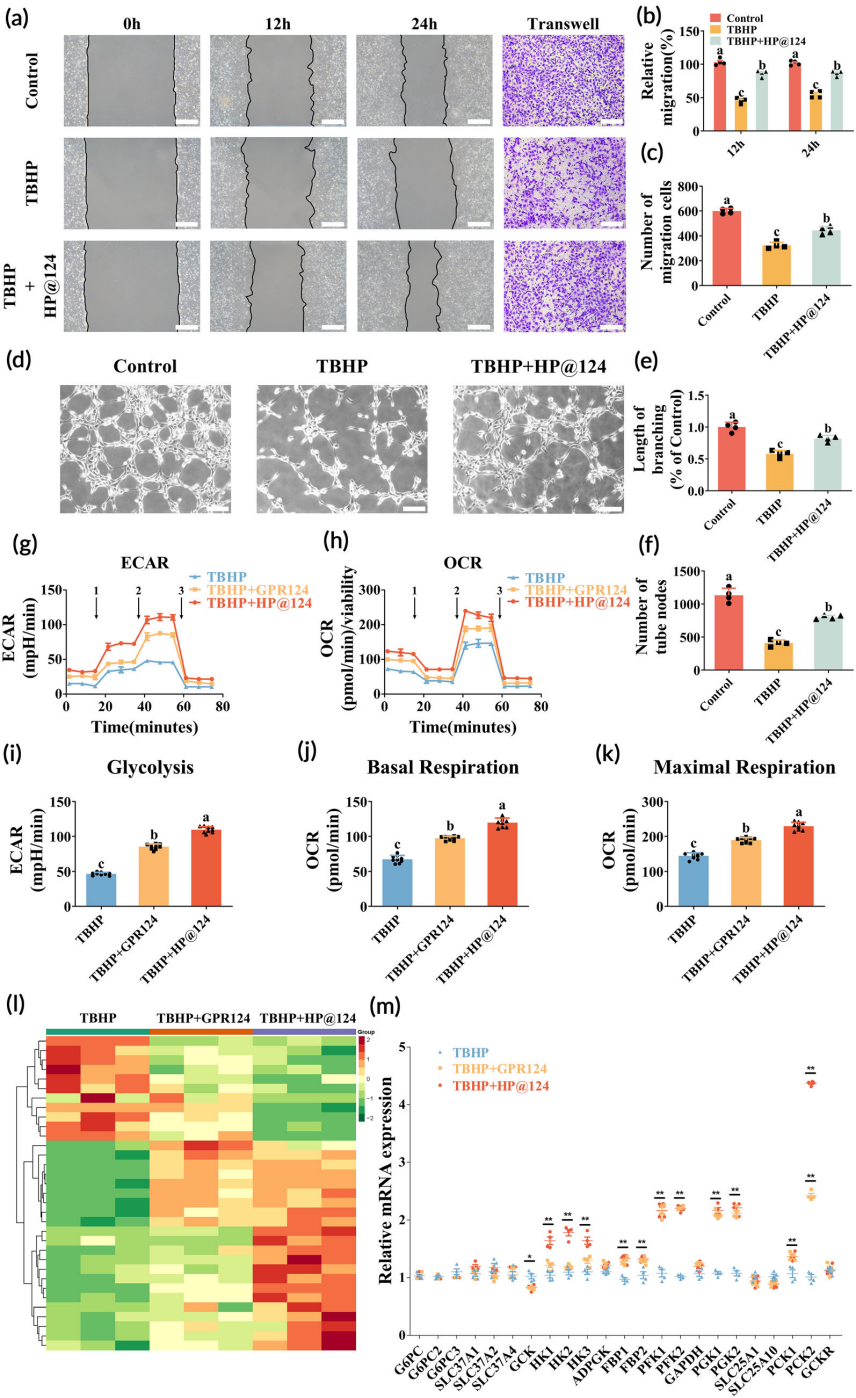
Role of heparin‐poloxamer (HP)@124 hydrogel on migration, angiogenesis, and energy metabolism of endothelial cells (ECs). (a) The horizontal and vertical migration of human cerebral microvascular endothelial cell line (HCMEC)/D3s tested by wound healing experiment and transwell experiment, respectively. Scale bar = 100 μm. (b,c) Quantification of relative horizontal migration after 24 h and vertical migration cell count (normalized to control values), values with different superscripts are significantly different (*p* < 0.05, one‐way analysis of variance (ANOVA) test, *n* = 4). (d) Tube formation images of human umbilical vein endothelial cell (HUVECs) in each group. Scale bar = 100 μm. (e,f) Quantification of relative branch length and count of nodes, values with different superscripts are significantly different (*p* < 0.05, one‐way ANOVA test, *n* = 4). (g,h) Extracellular acidification rate (ECAR) verified by glycolysis stress experiment (injection 1 = glucose, injection 2 = oligomycin, injection 3 = 2‐DG), oxygen consumption rate (OCR) level as verified by mitochondrial stress test (injection 1 = oligomycin, injection 2 = carbonyl cyanide 4‐(trifluoromethoxy)phenylhydrazone (FCCP), injection 3 = antimycin A/rotenone) using seahorse instrument. Detections were performed every 5 min per time point for each condition. (i) Quantification of glycolysis from one‐time point in glycolysis stress test, values with different superscripts are significantly different (*p* < 0.05, one‐way analysis of variance (ANOVA) test, *n* = 9). (j,k) Quantification of basal respiration and maximal respiration for each time point using mitochondrial stress test, values with different superscripts are significantly different (*p* < 0.05, one‐way ANOVA test, *n* = 9). (l) Heatmap of RNA sequencing of three groups (tert‐butyl hydroperoxide [TBHP], TBHP + G protein‐coupled receptor 124 [GPR124], TBHP + HP@124). (m) Real‐time PCR analysis of genes associated with glucose metabolism (*p < 0.05, **p < 0.01, one‐way ANOVA test, *n* = 4).

To further investigate the potential role of HP@124 hydrogel on rebuilding BSCB, we use HUVECs, and HCMEC/D3s, induced by TBHP to simulate the microenvironment post‐SCI. Wound healing and migration experiments indicated that HP@124 hydrogel notably elevated the horizontal and vertical migration of HCMEC/D3 after injury (Figure 3a–c). In addition, the angiogenesis of HUVECs after injury was remarkably raised by HP@124 hydrogel, as demonstrated by the number of tube nodes and the length of branching (Figure 3d,f).

Restoring the energy of cells post‐SCI could promote the prognosis of SCI.^27^ Herein, we first demonstrated that facilitating the glucose energy metabolism of ECs by GPR124 could also partially rebuild the BSCB post‐SCI. Accordingly, measurement was performed to investigate if GPR124 mediated the glucose metabolic alteration (Figure 3g,h). We found that glycolysis detected by ECAR of cells was promoted by treatment with GPR124, and HP@124 amplifies this effect (Figure 3i). Moreover, GPR124 markedly increased OCR, a biomarker of oxidative phosphorylation. Besides, basal and maximal respirations were significantly increased upon GPR124 addition, among which the HP@124 group improved most (Figure 3j,k).

To obtain insight into the mechanism by which HP@124 acts on HCMEC/D3s, we performed mRNA sequencing analysis of HCMEC/D3s cultivated on different substrates for 3 days (Figure 3l). Interestingly, the GO analysis among the three groups implied a strong correlation of differently expressed genes (DEGs) with the metabolic process (Figure S2A). KEGG pathway analysis demonstrated that DEGs were also enriched for functional annotations relating to the metabolic pathway (Figure S2B), which were consistent with the effect of GPR124 mentioned above.

Furthermore, the expression of key genes, especially several respiratory enzymes associated with glucose metabolism, was evaluated to verify the mechanism underlying the ameliorated energy metabolism. Compared with the TBHP group, GPR124 and HP@124 group exhibited an obvious effect on glucose metabolism‐associated genes. Among all tested genes, the expression of phosphoenolpyruvate carboxykinase 2 (PCK2) elevates the most, which is consistent with the sequencing result (Figure 3m). PCK2, as two isoforms of phosphoenolpyruvate carboxykinase (PEPCK), the key gluconeogenic enzyme, could promote cell metabolic flexibility to resist energy stress, modulate gluconeogenesis, tricarboxylic acid (TCA) cycle function, and even glycolysis.^28^ Collectively, our results demonstrated that HP@124 hydrogels may improve the energy metabolism effect of HCMEC/D3s and protect them from energy exhaustion by elevating the PCK2 expression, finally contributing to the regeneration of BSCB caused by SCI.

### 3.4 heparin‐poloxamer@124 hydrogel attenuated blood‐spinal cord barrier permeability and protected blood‐spinal cord barrier integrity by preventing the loss of tight junction proteins post‐SCI

**FIGURE 4:**
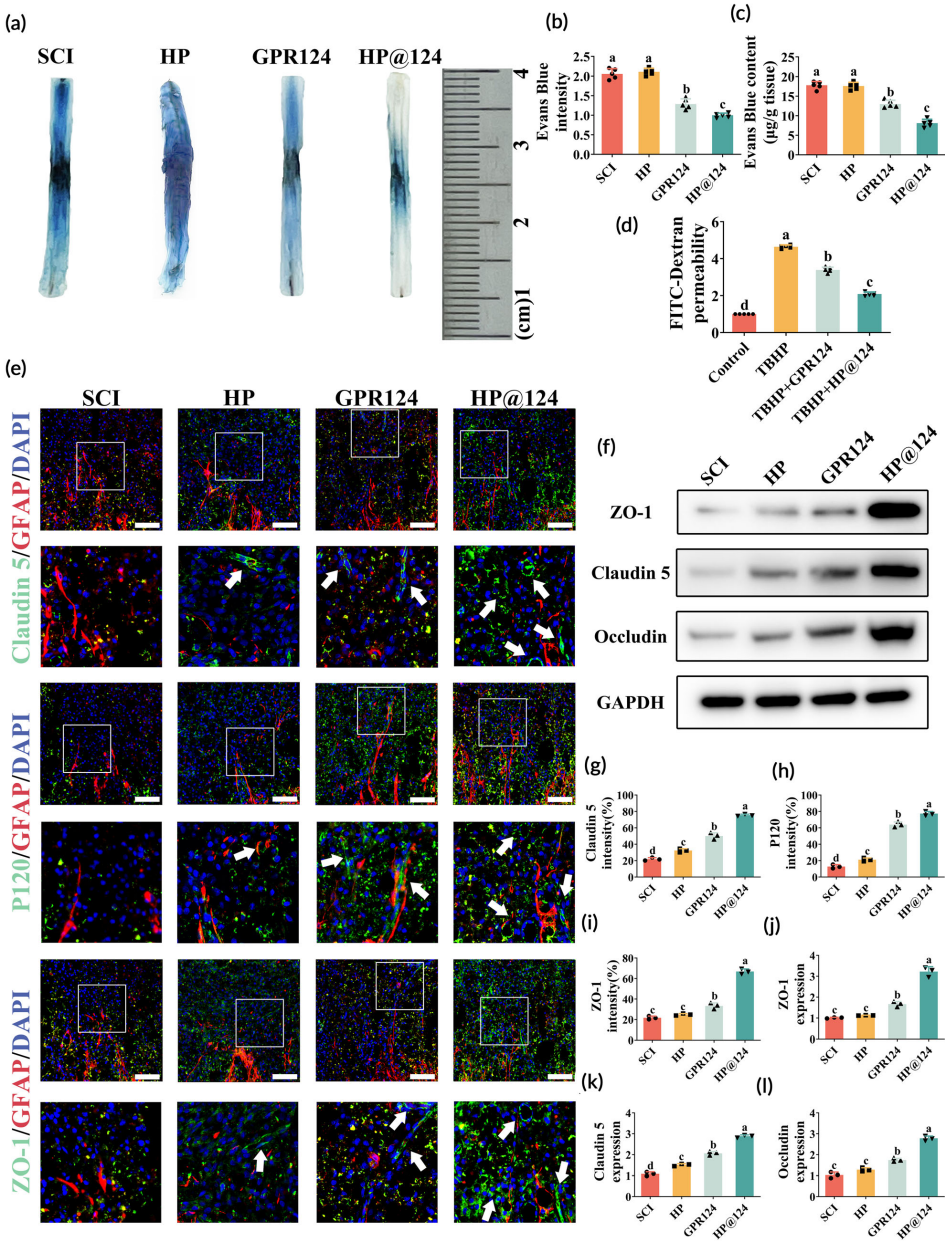
Heparin‐poloxamer (HP)@124 hydrogel inhibits the raise of blood‐spinal cord barrier (BSCB) permeability through restraining loss of tight junctions (TJ) proteins post‐spinal cord injury (SCI) in vivo. (a) Evans Blue (EB) staining permeability at three days post‐SCI. (b) Quantification of the EB's intensity of lesion site (%), values with different superscripts are significantly different (*p* < 0.05, one‐way analysis of variance (ANOVA) test, *n* = 5). (c) Quantification of the EB's content of the lesion site (μg g^−1^), values with different superscripts are significantly different (*p* < 0.05, one‐way ANOVA test, *n* = 5). (d) Barrier capacity of human cerebral microvascular endothelial cell line (HCMEC)/D3s tested by FITC‐dextran leakage, values with different superscripts are significantly different (*p* < 0.05, one‐way ANOVA test, *n* = 5). (e) The co‐immunofluorescence of Glial fibrillary acidic protein (GFAP) (red) and TJ proteins (green) including zonula occludens‐1 (ZO‐1), claudin 5 and P120 at 7 days post‐SCI in each group. White arrows mark tubular structures. Scale bar = 200 μm. (g–i) Quantification results of TJ proteins positive region, values with different superscripts are significantly different (*p* < 0.05, one‐way ANOVA test, *n* = 3). (f,j–l) Western blot analysis of ZO‐1, claudin 5, and occludin at 7 days post‐SCI and the related quantification results, values with different superscripts are significantly different (*p* < 0.05, one‐way ANOVA test, *n* = 3).

**FIGURE 5:**
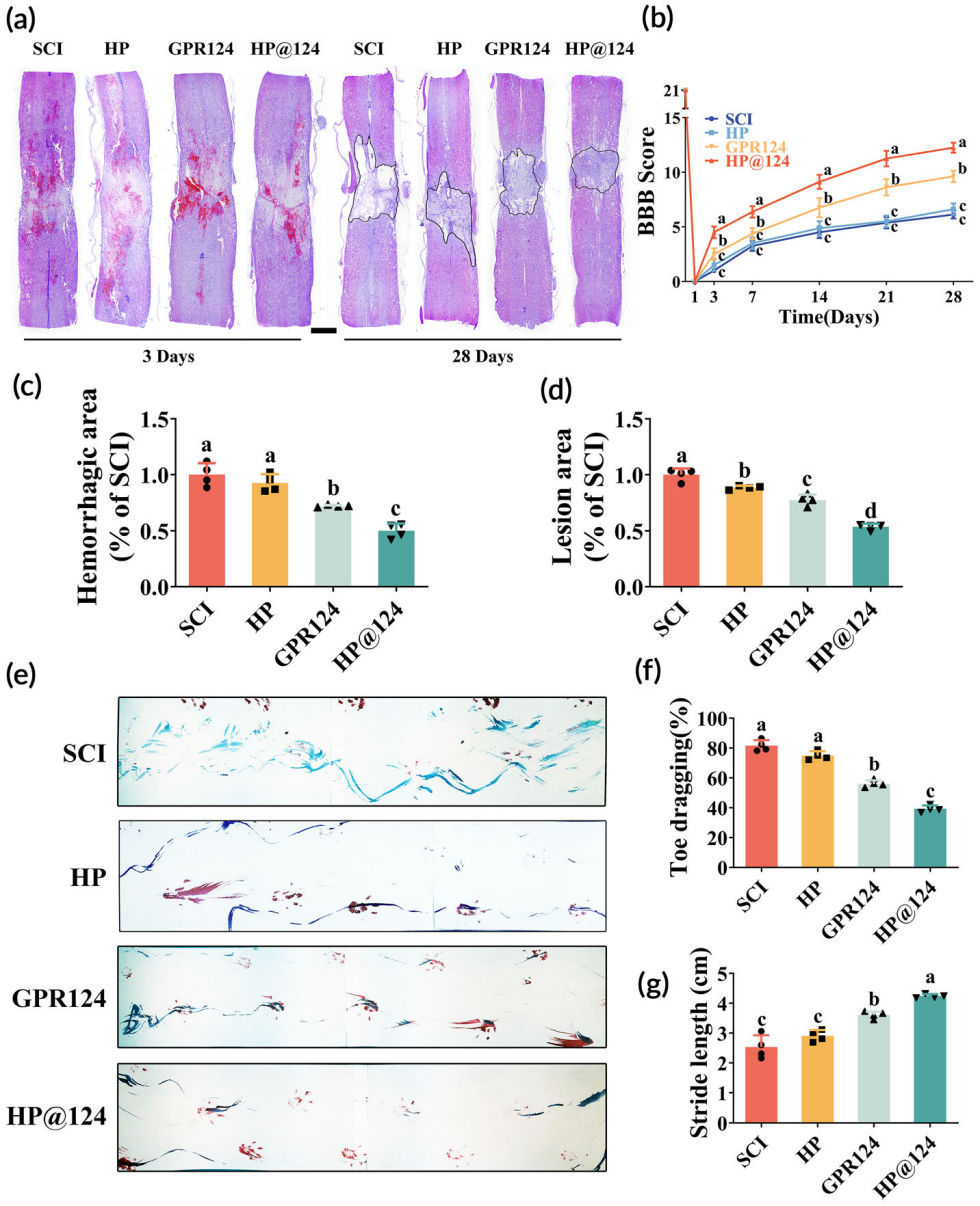
Heparin‐poloxamer (HP)@124 hydrogel ameliorates pathology and motor function post‐spinal cord injury (SCI). (a) Images of H&E staining at 3 and 28 days post‐SCI. Scale bar = 1 mm. (b) The BBB locomotion scores in each group, values with different superscripts are significantly different (*p* < 0.05, two‐way analysis of variance (ANOVA) test, *n* = 8). (c,d) Quantification of the lesion and hemorrhage area of the spinal cord from B, values with different superscripts are significantly different (*p* < 0.05, one‐way ANOVA test, *n* = 4). (e) Footprint analysis of each group. (f, g) Quantification of the ratio of toe dragging and stride length from f, values with different superscripts are significantly different (*p* < 0.05, one‐way ANOVA test, *n* = 4).

To evaluate whether the favorable effects of HP@124 were involved in the BSCB conservation in vivo, we detected the degrees of extravasation post‐SCI. EB staining was decreased in the free GPR124 group and HP@124 group contrasted to the HP group and SCI group at 72 h post‐injury, implying maintenance of BSCB integrity (Figure 4a,b). Besides, the EB content was sequenced as the HP@124 < GPR124 < HP ≈ SCI group (Figure 4c). Similar results in H&E staining indicated that the HP@124 group has minimum hemorrhage area at 3 days post‐SCI (Figure 5b,d).

Moreover, in vitro BSCB model composed of a monolayer of HCMEC/D3s showed similar results. The addition of GPR124 significantly attenuated the TBHP‐induced endothelial hyperpermeability to dextran (Figure 4d). Taken together, compared with the SCI, HP, and free GPR124 group, the HP@124 group showed more benefit in BSCB protection post‐SCI, both in attenuating permeability and protecting integrity.

The TJ disruption between BSCB may explain the increased permeability of the BSCB. We investigated whether HP@124 hydrogel diminishes the rised permeability by preventing the loss of TJ proteins post‐SCI. Immunofluorescence showed that the fluorescence intensity of TJs, including Claudin 5, delta 1 Catenin (P120), and ZO‐1, was greatly elevated in the HP@124 group, as compared to GPR124, HP, and SCI groups (Figure 4e). In addition, compared with the HP‐only and GPR124‐omly group, the HP@124 group had more positive fluorescent areas of TJs (Figure 4g,i) with more extended tubular vascular structures (Figure 4e, white arrow mark). Furthermore, WB was used to examine the expression of the TJ proteins in spinal lysates, which showed similar results. HP@124 group indicated notably higher levels of ZO‐1, Occludin, and Claudin 5 at 7 days post‐SCI contrasted to SCI group. In addition, TJ proteins exerted a mildly elevated level in HP and GPR124 group, while still lower than HP@124‐treated group (Figure 4f,j–l), revealing that GPR124 restrains loss of TJ proteins post‐injury, meanwhile, HP hydrogel synergistically prolongs and amplifies its effect.

### 3.5 Ameliorated pathology and motor function post‐spinal cord injury by heparin‐poloxamer@124 hydrogel

Although we find that GPR124 could restore the function of BSCB post‐SCI in the acute phase, considerable research regarded it as preventing the disruption of BSCB failed to promote motor recovery in the chronic phase post‐SCI.^29^ Hence, we performed extensive pathological and functional evaluations to assess the motor neuron restoration in the chronic phase post‐SCI. To evaluate whether the GPR124 and HP@124 hydrogels could improve functional motor restoration in rats with SCI, The BBB locomotion scores along with the footprint test were employed in a functional evaluation (Figure 5b,e). As the progress of SCI, the injured rats exhibited flaccid paralysis initially, and then restored appropriately with recovery time went by (Figure 5b). Following 14 days post‐injury, the scores of rats treated with free GPR124 (average BBB score = 7) and HP@124 (average BBB score = 10) were higher than those of rats in HP group (average BBB score = 5) and SCI group (average BBB score = 5). On day 21, the BBB scores of each group improved steadily. Compared with all other groups, the significantly high scores in the HP@124 group (average BBB score = 12) were observed on day 28 post‐injury. Moreover, the scores of GPR124‐only treated group also showed remarkable difference in the chronic phase of SCI as compared with SCI group, along with almost no difference between the scores of HP and SCI group (Figure 5b). On day 28 post‐SCI, the footprint test demonstrated that rats in the HP@124 group had an extremely steady posterior limb (blue ink) imprint, moderate synchronization, and few stumbles. Whereas rats in free GPR124 groups showed intermittent dragging behavior. Yet, contrasted to the HP and SCI group, the stride length extended in this group, indicating that the ankle, knee, and hip joints of the posterior limbs of rats recovered in a more excellent manner and can move substantially (Figure 5e–g). We demonstrate that both the free GPR124 and HP@124 hydrogels treatment could promote the functional motor recovery of rats post‐SCI, and HP@124 hydrogels exhibited the optimal treatment effect on SCI rats contrasted to other groups.

H&E staining revealed the histological structure of spinal cord on 28 days post‐SCI (Figure 5a). Compared with the SCI group with an obvious cavity, the lesion region of the damaged spinal cords medicated with HP, free GPR124 and HP@124 significantly shrank (Figure 5c,d). Moreover, the volume of whole bladders 28 days post‐SCI indicated the best recovery of urinary retention in the HP@124 group (Figure S3A&B).

Together, we indicate that HP@124 hydrogel could recover the function of locomotion in the chronic phase of SCI.

### 3.6 Accelerated axon regeneration and reduced fibrotic scar formation by heparin‐poloxamer@124 hydrogel

**FIGURE 6:**
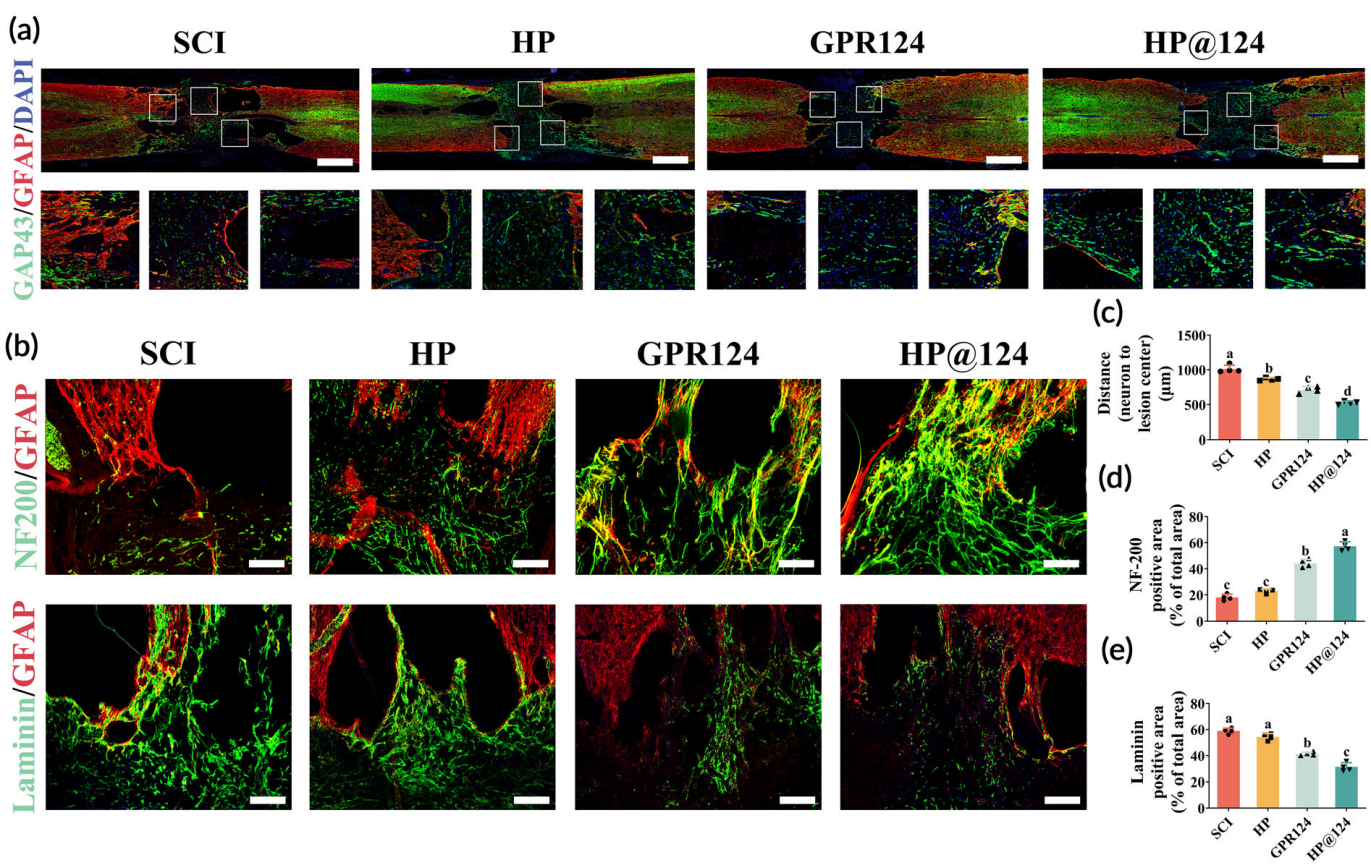
Heparin‐poloxamer (HP)@124 hydrogel enhances axonal regeneration and reduces fibrotic scar tissue formation post‐spinal cord injury (SCI). (a) The co‐immunofluorescence of glial fibrillary acidic protein (GFAP) (red) and GAP43 (green) at 28 days post‐SCI. Scale bar = 500 μm. (b) Immunofluorescence images of fibrotic scar or neurofilament (green) and astrocytes (red) at 28 days post‐SCI. Scale bar = 200 μm. (c) Quantification of distance from neurons to epicenter of lesion, values with different superscripts are significantly different (*p* < 0.05, one‐way analysis of variance (ANOVA) test, *n* = 4). (d) Quantitative analysis of NF‐200 positive axon region in the lesion, values with different superscripts are significantly different (*p* < 0.05, one‐way ANOVA test, *n* = 4). (e) Quantitative analysis of laminin‐positive fibrotic scars region in the lesion, values with different superscripts are significantly different (*p* < 0.05, one‐way ANOVA test, *n* = 4).

Next, we evaluate the effect on various aspects on day 28 post‐SCI treating with HP@124 hydrogel. Restoration of nerve conduction, especially axonal regeneration, is one of the potential approaches for the regeneration of SCI. The effect of axonal rehabilitation by HP@124 hydrogel was examined by performing immunofluorescence. First, Immunofluorescence was employed to calculate the distance between the closest neurons (GAP43‐positive cells) and the epicenter using Growth associated protein‐43 (GAP43) (Figure 6a). Compared to the SCI group, the gap in the wounded spinal cords treated with free GPR124 and HP@124 shrunk significantly, showing that both treatments might enhance axon regeneration, with the HP@124 hydrogel having the greatest effect on enhancing the axonal restoration post‐SCI. In addition, the HP group also showed a mild effect on shortening the distance between the broken ends of spinal cord (Figure 6C). Furthermore, glial fibrillary acidic protein (GFAP) (red) and Neurofilament heavy polypeptide (NF‐200) (green) double immunofluorescence staining was conducted to investigate the epicenter of the injured spinal cord and the expansion of the neurofilaments (Figure 6b). In the SCI‐only and HP‐only group, rare NF‐200 positive axons were detected in the region, but the free GPR124 group displayed significant punctiform or tubular NF‐200 positive axons. In addition, HP@124 therapy significantly promotes the regeneration of neurofilaments, which could penetrate the glial scar barrier and exhibit expanded axons (Figure 6d).

Scars proliferate rapidly after the acute phase of SCI, especially fibrous scar, which impedes the regeneration of axons.^30^ Abundant laminin‐positive fibrous scars were centralized in the injured site and the damaged boundary post‐SCI (Figure 6b). While the laminin‐positive fibrotic scarring area was significantly limited following HP@124 hydrogel treatment. Contrasted to the HP and SCI group, decreased laminin‐positive region also appears in the treatment of free GPR124 (Figure 6e).

In conclusion, our results indicated that HP@124 hydrogel might stimulate axon growth and inhibit the establishment of fibrous scars.

### 3.7 Heparin‐poloxamer@124 hydrogel promoted remyelination and attenuated inflammation response post‐SCI

**FIGURE 7:**
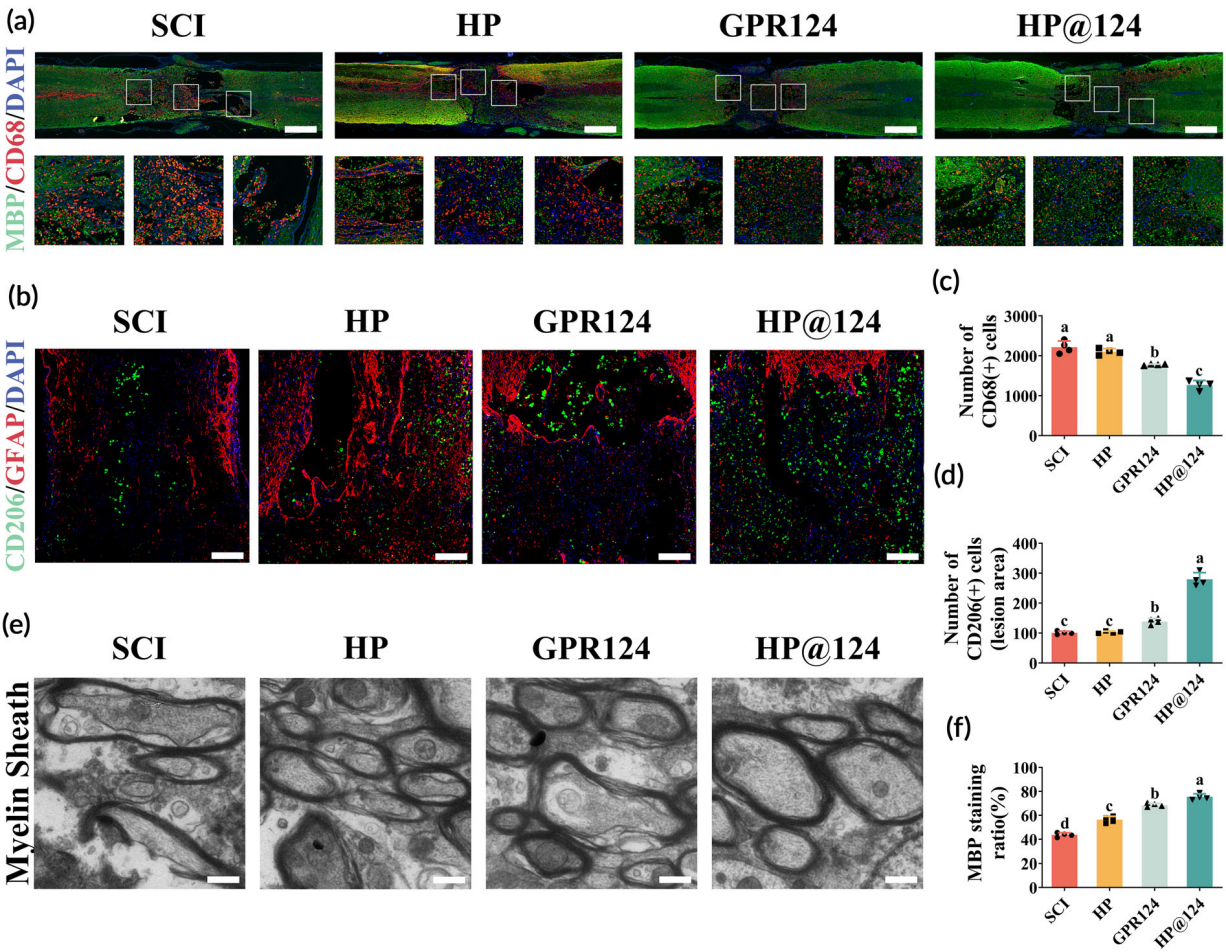
Heparin‐poloxamer (HP)@124 mitigates inflammatory reactions and enhances remyelination post‐spinal cord injury (SCI). (a) myelin basic proteins (MBP) (green) and CD68 (red) immunofluorescence of spinal cord at 28 days post‐injury. Scale bar = 500 μm. (b) The co‐immunofluorescence of glial fibrillary acidic protein (GFAP) (red) and CD206 (green) at 28 days post‐injury. Scale bar = 200 μm. (c) Quantification of number of CD68‐positive cells from A, values with different superscripts are significantly different (*p* < 0.05, one‐way analysis of variance [ANOVA] test, *n* = 4). (d) Quantification of number of CD206 positive cells from B, values with different superscripts are significantly different (*p* < 0.05, one‐way ANOVA test, *n* = 4). (e) the Transmission electron microscopy (TEM) images of myelin sheath at 28 days post‐injury. Scale bar = 2 μm. (f) Quantification results of MBP staining ratio from A, values with different superscripts are significantly different (*p* < 0.05, one‐way ANOVA test, *n* = 4).

The relationship between remyelination and inflammation has been widely reported.^31^ The appropriate level of inflammation could promote remyelination. To investigate the myelin sheath destruction and regeneration, each group's myelin basic proteins (MBPs) and CD68 were evaluated using immunofluorescence (Figure 7a). The findings revealed that the MBP protein expression sharply dropped post‐SCI but was remarkably increased by the HP, GPR124, and HP@124 treatment (Figure 7f). The least number of CD68‐positive cells in the HP@124 group may be responsible for its best remyelination (Figure 7c). Meanwhile, we also observed the increased number of CD206 positive cells which indicated the anti‐inflammatory macrophage phenotypes in the GPR124 and HP@124 group, as compared with the HP and SCI group (Figure 7b,d). These findings may also explain the GPR124 and HP@124 groups' prominent effect on remyelination and even on the improved microenvironment. Furthermore, an electron microscope showed that HP@124 treated group has the most intact and well‐organized myelin sheath with abundant microtubules in the axon (Figure 7e), similar to the immunofluorescence results.

## 4 DISCUSSION

The concept of neurovascular units has attached sufficient attention of researchers in recent years, which consists of BSCB and related neurons.^32^, ^33^ However, therapies of SCI focused more on the regeneration of neuron while losing the sight of the BSCB rebuild, which is also considered a potential therapy that promotes the function recovery of SCI.^23^ Herein, our study aimed at exploring the potential targets in the process of development and maturation of BSCB, which could provide a new perspective for the rebuild of BSCB as well as the recovery of SCI.

In our study, we demonstrated the effect of GPR124 on BSCB reconstruction post‐SCI. GPR124, known as TEM5, was first discovered in the vasculature of human colorectal cancer,^34^ then was found to be required for the establishment of the blood–brain barrier.^7^ However, it is unknown whether it helps to restore BSCB post‐SCI in adult animals. Herein, we indicated that with time going by post‐SCI within 1 week, the expression of GPR124 protein decreased over time, similar to the expression of TJ proteins. We assumed this might be an important reason for BSCB regeneration failure post‐SCI. Accordingly, our study intended to use the recombinant protein of GPR124 to elevate the low expression level of GPR124 post‐SCI, and eventually stimulated the reconstruction of BSCB.

The medicinal usage of free proteins to remedy SCI is restricted owing to their short half‐life, fast degradation, and adverse outcomes at high concentrations.^35^ Post‐SCI, MMP2, and MMP9 were increased obviously,^36^ which would further accelerate protein degradation. Accordingly, measures must be taken to maintain GPR124's biological activity to protect the disrupted BSCB for a longer‐lasting time. Considering that GPR124 could directly interact with glycosaminoglycans including heparin.^10^ Combined with previous works, we designed an injectable, thermosensitive hydrogel with a constant release of GPR124 to restrain BSCB disruption and encourage functional restoration and motor rehabilitation post‐SCI. Our study is the first to reconstruct BSCB in the early stage of SCI and ultimately promote the long‐term recovery of SCI from the perspective of developmental biology of BSCB.

In recent years, HP hydrogel has been synthesized and used in different fields, such as SCI regeneration,^12^, ^37^ peripheral nerve regeneration,^38^ and vascular anastomosis.^39^ Herein, our research focused more on the function of GPR124 and the synergistic effect of GPR124 and HP hydrogel, which broadens the application of HP hydrogel in bridging proteins and provides new possibilities for the diversified use of HP hydrogel. Compared to other delivery strategies, HP@124 hydrogel had significant advantages. First, HP@124 hydrogel had various properties, including thermo‐sensitivity, biomechanical property, and injectability, which provided a favorable environment for injured tissue, filling the injured tissue cavities and limiting inflammation. Second, HP@124 hydrogel could efficiently load GPR124 and permit sustained release, which greatly compensates for the limited effects of HP hydrogel on the acute phase treatment of SCI. Last but not the least, P is an FDA‐approved and highly safe pharmacological ingredient in our hydrogel, providing a promising potential for clinical translation. As a result, the synergistic effect of HP hydrogel and GPR124 has contributed to both the acute phase treatment and the long‐term recovery of SCI.

At a molecular level, oxidative stress and free radicals play an important part in BSCB disruption.^40^, ^41^ It is also one of the reasons why we used TBHP to mimic the microenvironment of SCI in vitro. Two different type of ECs both induced by TBHP were used here to simulate the disrupted BSCB and the adverse microenvironment post‐SCI in order to clarify the in vitro effect of HP@124 hydrogel on BSCB. We demonstrated that GPR124 could rescue the horizontal and vertical migration abilities of the injured ECs to varying degrees, which may be due to the directional arrangement of blood vessels in BSCB. Moreover, GPR124 could also promote the angiogenesis of injured ECs, which is the key to the reconstruction of BSCB.

As an orphan receptor of G protein, currently, there are no ligands or agonists that fully adapt to GPR124. However, in the studies of GPR124 related to the function of vascular regeneration and adhesion, its mechanism may not lie on the function of G protein receptor.^42^, ^43^ Hence, transcriptome analysis was carried out here to further confirm the mechanism of HP@124 hydrogel on HCMEC/D3s. Our bioinformatics analysis revealed that the effects on the microvascular endothelial cells were related to the process of metabolism, of which the main way is glucose metabolism, including glycolysis and oxidative phosphorylation. While glycolysis plays a major role, oxidative phosphorylation also contributes significantly to the production of total adenosine triphosphate (ATP).^44^, ^45^

Seahorse test showed that the elevated metabolic level with the treatment of HP@124 rescues the poor state of ECs, and may promote migration and restore TJs. However, further research is required to verify the association between energy metabolism and BSCB restoration. We demonstrated that PCK2 might be the switch of this restoration of energy metabolism, which needs more testing to confirm. Whether the elevated energy metabolism will influence other cells, such as neuronal cells and astrocytes, remains unknown. Together, these findings are of great significance in helping explain the independent mechanism of GPR124.

Spinal cord crushing injury model of rats was established here to verify the effect of HP@124 hydrogel on the reconstruction of BSCB in the early stage post‐SCI in vivo. The volume of the injected contents is referenced to the previous research.^11^, ^12^, ^46^, ^47^ Moreover, the modulus of HP hydrogel containing 500 ng GPR124 is similar to spinal cord.^48^, ^49^ As a result, the HP@124 hydrogel would not induce compression on the spinal cord. The initial release of hydrogel ensured the initial concentration of GPR124, and the concentration was maintained by the subsequent sustained release at a stable level. The significant effect of the sustained release of GPR124 is also due to the protection of heparin, which delayed the degradation of GPR124. However, the dosage of GPR124 used in vivo requires further exploration.

BSCB breakdown post‐SCI occurs within 5 min,^50^ exhibiting structural disturbance and high permeability, and peaks within 1 week, while the restoration of integrity takes at least 2 weeks and the functional recovery of barrier takes more times.^51^, ^52^ The integrity of the BSCB is essential for maintaining spinal cord function.^53^ However, due to the obvious edema of spinal cord tissue and massive infiltration of blood cells on the first‐day post‐injury, we chose 3 days post‐injury as our sample harvest time to detect the integrity of BSCB in each group.^54^ Among the several cellular mechanisms accountable for the long‐term rise in BSCB permeability post‐SCI is the disruption of TJs.^55^ Considering the delayed recovery time of functional recovery of BSCB, we select 7 days post‐injury as our sample harvest time to test the protein level of TJs in each group.^24^

In the acute phase of SCI, simple HP hydrogel had limited effects on the rebuild of BSCB, while the addition of GPR124 and the synergistic effect between GPR124 and HP hydrogel enable this delivery system to provide more BSCB protection, including structural and functional repair, which is detected by EB staining and the protein level of TJs, respectively.

Treatment strategies for the long‐term recovery of SCI include controlling inflammation,^56^ remyelination,^57^ altering scar formation,^58^ and axon regeneration.^59^ Herein, multiple integrated in vivo evidence was gathered, including functional measures, pathological analysis, and immunofluorescence staining.

Balancing the relationship between inflammation and remyelination is important for the regeneration of SCI. None or immoderate inflammatory response will restrict remyelination.^31^, ^60^, ^61^ Herein, HP@124 groups could significantly enhance remyelination, suppress the immoderate inflammatory reaction which may be associated with remyelination, and help axon reconstruction. Partially repaired BSCB, which prevents excessive inflammatory cells from entering the lesion, may be the reason.^62^ Meanwhile, the ECs that restored energy metabolism may also result in the transition of macrophages towards a more pro‐regenerative/anti‐inflammatory phenotype.^63^

Furthermore, the inhibitory microenvironment for axonal regrowth in the chronic phase of SCI is mainly resulted by fibrotic scar.^64^, ^65^ We revealed that the formation of fibrotic scar was notably suppressed by HP@124 hydrogels. Meanwhile, the researchers' attention often focus on axon regeneration and motor function recovery due to the potential for clinical translation.^66^ HP@124 group revealed obvious motor rehabilitation based on BBB scores at 28 days post‐SCI contrasted to the GPR124 group. And HP@124 group notably ameliorated the axonal reconstruction by motivating the GAP43 and neurofilament, confirming the contribution to axonal regeneration and neuron protection.

Although there are still differences between rodents and humans in spinal cord size, anatomical structure, function, and inflammatory response,^67^ the rat SCI model is still the most commonly used and accepted experimental animal model at present.^68^, ^69^ Meanwhile, we also note that during the injury, the dura of rat is thin and easily disrupted by compression, resulting in the direct in situ injection of HP@124 hydrogel. Clinically, there are also cases of SCI with a relatively intact dura. In these cases, intrathecal injection of HP@124 during decompression surgery could be performed in the consideration of the safety of intravenous injection of P, providing the basis for further effective clinical translational research.

## 5 CONCLUSION

In summary, we verified the decrease of GPR124 post‐SCI and the important role of recombinant GPR124 protein in restoring BSCB in vivo and in vitro. Meanwhile, a thermosensitive hydrogel has been prepared to conserve GPR124 bioactivity and restraint its release. The HP@124 hydrogel notably ameliorated the SCI regeneration when administered via in situ injections in vivo. HP@124 hydrogel exerted neuroprotective effects and created promising conditions for functional rehabilitation due to its BSCB hyper‐permeability attenuation, TJ disruption suppression, fibrotic scars restraintion, inflammatory response attenuation, remyelination and axonal reconstruction by protecting BSCB from disruption, restoring TJ proteins and energy metabolism. Combined with the moderate regeneration effects post‐SCI treated by free GPR124 alone, HP@124 hydrogel could facilitate and prolong GPR124 delivery to the lesion site.

Together, the marked effect of GPR124 in BSCB restoration at the early stage of SCI and the neuro‐preservation benefit of this delivery strategy in easy fabrication, fast gelation, good biocompatibility, and biodegradability give rise to a clinically feasible therapeutic manner for patients with SCI.

## AUTHOR CONTRIBUTIONS

**Jiawei Shu:** Conceptualization (lead); data curation (lead); formal analysis (lead); investigation (lead); methodology (lead); project administration (lead); writing – original draft (lead). **Chenggui Wang:** Conceptualization (equal); data curation (equal); formal analysis (equal); investigation (equal); methodology (equal); writing – original draft (equal). **Yiqing Tao:** Conceptualization (equal); formal analysis (equal); funding acquisition (equal); investigation (equal); methodology (equal); writing – original draft (equal). **Shaoke Wang:** Formal analysis (equal); methodology (equal); software (equal). **Feng Cheng:** Software (equal); supervision (equal); writing – review and editing (equal). **Yuang Zhang:** Formal analysis (equal); writing – review and editing (equal). **Kesi Shi:** Conceptualization (equal); supervision (equal). **Kaishun Xia:** Conceptualization (equal); supervision (equal). **Ronghao Wang:** Conceptualization (equal); supervision (equal). **Jingkai Wang:** Conceptualization (equal); supervision (equal). **Chao Yu:** Supervision (equal). **Jiangjie Chen:** Methodology (equal). **Xianpeng Huang:** Methodology (equal). **Haibin Xu:** Methodology (equal). **Xiaopeng Zhou:** Methodology (equal). **Haobo Wu:** Writing – review and editing (equal). **Chengzhen Liang:** Writing – review and editing (equal). **Qixin Chen:** Conceptualization (equal); funding acquisition (equal). **Shigui Yan:** Funding acquisition (equal); supervision (equal). **Fangcai Li:** Funding acquisition (lead); supervision (lead).

## CONFLICT OF INTEREST STATEMENT

All authors declare that they have no conflict of interest.

## Supporting information


**Data S1:** Supporting Information.

## Data Availability

Data available on request from the authors.
