# Supplementary material for: Thermosensitive hydrogel‐based GPR124 delivery strategy for rebuilding blood‐spinal cord barrier
Source: Bioeng Transl Med. 2023 Jun 6;8(5):e10561. doi: 10.1002/btm2.10561 (PMC10486335; doi:10.1002/btm2.10561)
Supplement: Supplementary file 1 — Data S1: Supporting Information. [file BTM2-8-e10561-s001.docx]

**Inventory of Supplemental Information**

**I** Supplemental Figures and Legends

Figure S1, related to Figure 2;

Figure S2, related to Figure 3;

Figure S3, related to Figure 5.

**II** Supplemental Tables

Table S1, related to Figures 4, 6 and 7;

Table S2, related to Figures 1, 2 and 4;

Table S3, related to whole manuscript.

**I** Supplemental Figures and Legends


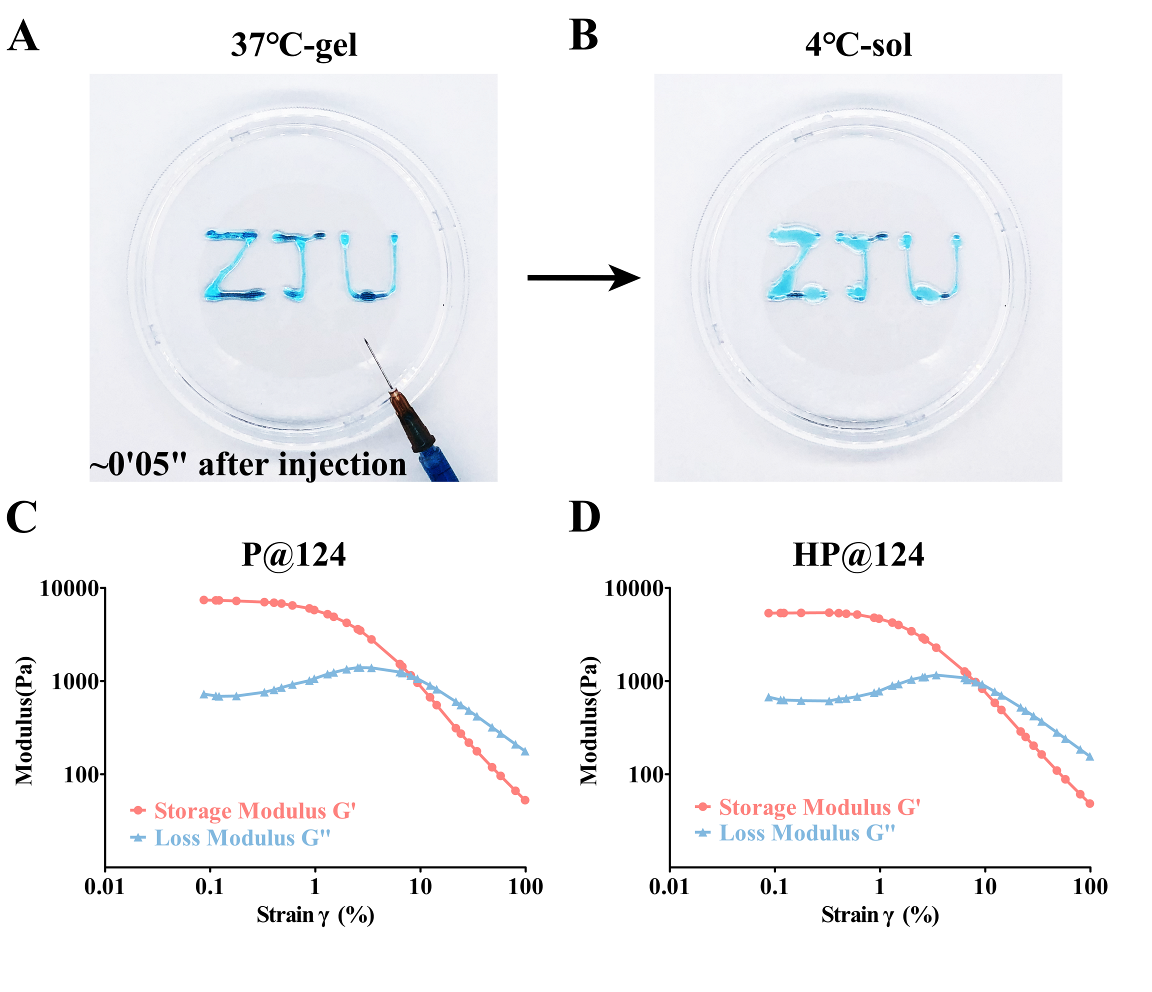


**Figure S1, related to Figure 2. HP@124 hydrogel Characterizations.**

(A-B) HP solution with blue dye (4℃) was injected into a 37℃ preheated cell culture dish by a 26-gauge syringe and quickly transited into gel, which maintained the shape of letters. And after transferred to 4℃ condition, the HP hydrogel again transited into solution. (C-D) Amplitude sweep of P@124 and HP@124 hydrogels displaying storage modulus (G′) and loss modulus (G″).


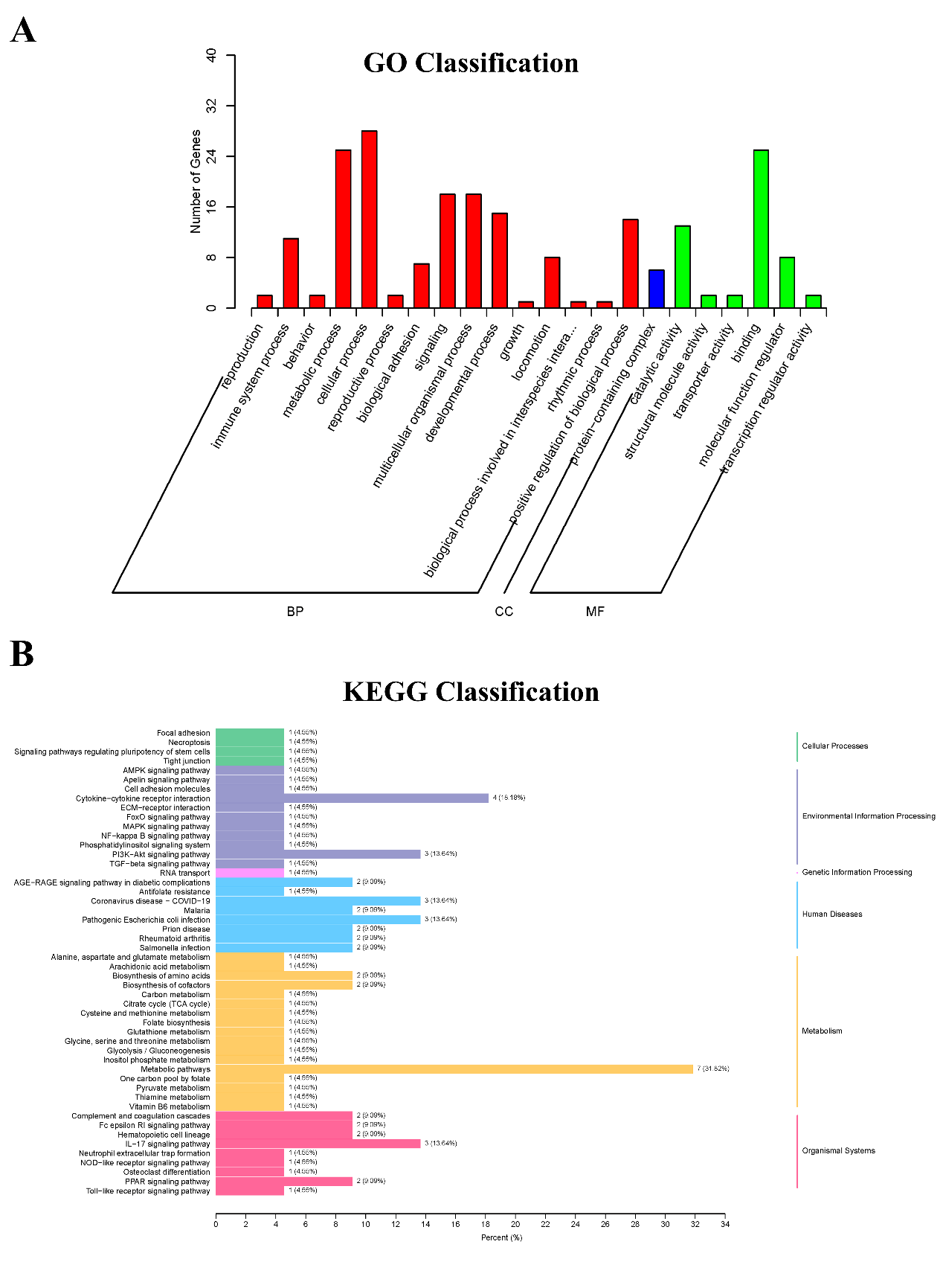


**Figure S2 Related to Figure 3. Role of HP@124 hydrogel on migration, angiogenesis and energy metabolism of ECs.**

(A) GO enrichment bar plots (metabolic process, cellular process and binding). (B) KEGG enrichment bar plots (metabolic pathways).


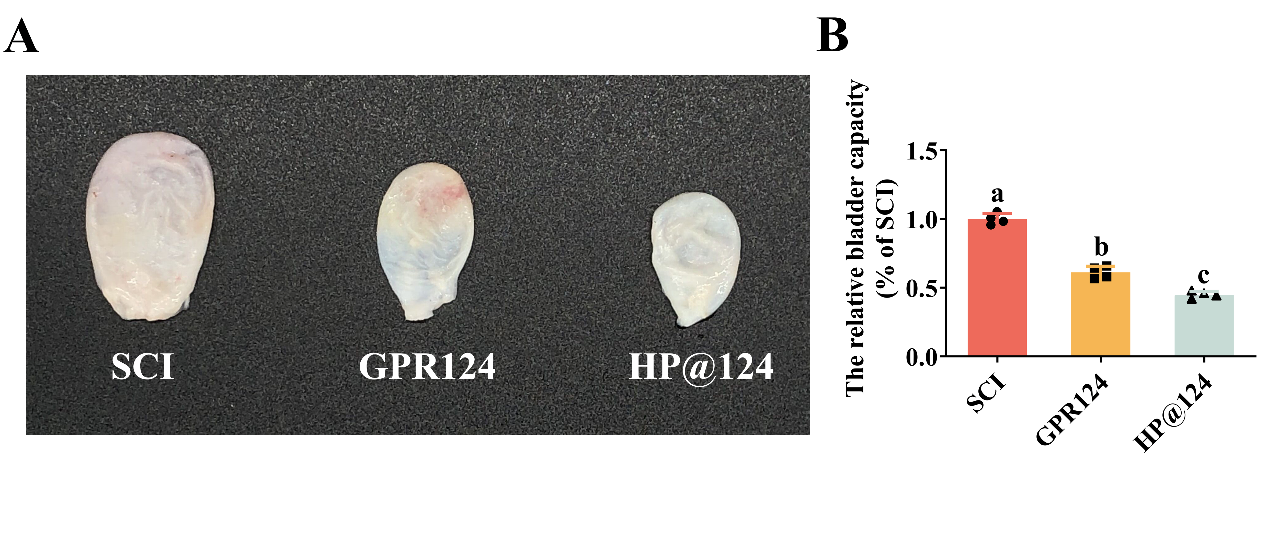


**Figure S3, related to Figure 5. HP@124 hydrogel ameliorates pathology and motor function post-SCI.**

(A) Images of the whole bladders of different groups. (B) Quantification of the relative volume of whole bladders, values with different superscripts are significantly different (*p* < 0.05, one-way ANOVA test, n=4).

**II** Supplemental Tables

**Table S1. Primary and secondary antibodies used for immunofluorescence, related to Figures 4, 6 and 7.**

| Antibodies | Species | Dilution | Supplier information | Catalog |
| --- | --- | --- | --- | --- |
| Anti-ZO1 tight junction protein (ZO-1) | Rabbit | 1:200 | Abcam | ab216880 |
| Anti-Claudin 5 | Rabbit | 1:250 | Abcam | ab15106 |
| Anti-Occludin | Rabbit | 1:250 | Abcam | ab216327 |
| Anti-delta 1 Catenin/CAS (P120) | Rabbit | 1:300 | Abcam | ab92514 |
| Anti-Growth associated protein-43 (GAP43) | Rabbit | 1:300 | Cell Signaling Technology | #8945 |
| Anti-Neurofilament heavy polypeptide (NF200) | Rabbit | 1:500 | Abcam | ab207176 |
| Anti-Neurofilament heavy polypeptide (NF200) | Rabbit | 1:500 | Abcam | ab8135 |
| Anti-Glial fibrillary acidic protein (GFAP) | Mouse | 1:500 | Sigma | G3893 |
| Anti-Glial fibrillary acidic protein (GFAP) | Goat | 1:500 | Abcam | ab53554 |
| Anti-Glial fibrillary acidic protein (GFAP) | Rabbit | 1:500 | Sigma | AB5804 |
| Anti-Glial fibrillary acidic protein (GFAP) | Rabbit | 1:500 | Abcam | ab7260 |
| Anti-Glial fibrillary acidic protein (GFAP) | Mouse | 1:400 | Cell Signaling Technology | #3670S |
| Anti-Laminin | Rabbit | 1:100 | Abcam | ab11575 |
| Anti-Myelin Basic protein (MBP) | Rabbit | 1:200 | Abcam | ab40390 |
| Anti-CD68 | Rabbit | 1:300 | Abcam | ab125212 |
| Anti-CD68 | Mouse | 1:300 | Abcam | ab955 |
| Anti-CD206 | Rabbit | 1:250 | Cell Signaling Technology | #24595S |
| Goat Anti-Mouse IgG H&L (Alexa Fluor® 488) | Goat | 1:500 | Abcam | ab150113 |
| Goat Anti-Mouse IgG H&L (Alexa Fluor® 555) | Goat | 1:500 | Abcam | ab150114 |
| Goat Anti-Rabbit IgG H&L (Alexa Fluor® 488) | Goat | 1:500 | Abcam | ab150077 |
| Goat Anti-Rabbit IgG H&L (Alexa Fluor® 555) | Goat | 1:500 | Abcam | ab150078 |
| Donkey Anti-Goat IgG H&L (Alexa Fluor® 555) | Donky | 1:500 | Abcam | ab150134 |
| Donkey Anti-Goat IgG H&L (Alexa Fluor® 488) | Donky | 1:500 | Abcam | ab150129 |

**Table S2. Primary and secondary antibodies used for Western blot, related to Figures 1, 2 and 4.**

| Antibodies | Species | Dilution | Supplier information | Catalog |
| --- | --- | --- | --- | --- |
| Anti-GPCR GPR124 | Rabbit | 1:250 | Abcam | ab67280 |
| Anti-ZO1 tight junction protein (ZO-1) | Rabbit | 1:500 | Abcam | ab216880 |
| Anti-Claudin 5 | Rabbit | 1:500 | Abcam | ab15106 |
| Anti-Occludin | Rabbit | 1:500 | Abcam | ab216327 |
| Anti-GAPDH | Rabbit | 1:10000 | Abcam | ab181602 |
| Goat Anti-Rabbit IgG H&L (HRP) | Goat | 1:500 | Beyotime Biotechnology | A0208 |
| Goat Anti-Mouse IgG H&L (HRP) | Goat | 1:500 | Beyotime Biotechnology | A0216 |

**Table S3. Abbreviation used in whole manuscript.**

| SCI | spinal cord injury |
| --- | --- |
| BSCB | blood-spinal cord barrier |
| GPR124 | G protein-coupled receptor 124 |
| TJ | tight junction |
| EC | endothelial cell |
| MMP | matrix metalloproteinase |
| FTIR | Fourier transform infrared spectroscopy |
| EB | Evans Blue |
| HE | Hematoxylin eosin |
| BBB | Basso-Beattie-Bresnahan |
| HCMEC/D3 | human cerebal microvascular endothelial cell line |
| HUVEC | human umbilical vein endothelial cell |
| TBHP | tert-butyl hydroperoxide |
| PCK2 | Phosphoenolpyruvate Carboxykinase 2 |
| FITC | fluorescein isothiocyanate isomer |
| DMEM | Dulbecco modified Eagle medium |
| PBS | phosphate buffer saline |
| PBST | phosphate buffer saline tween-20 |
| RIPA | radio immunoprecipitation assa |
| PMSF | phenylemthanesulfonyl fluoride |
| SDS | sodium dodecyl sulfate |
| PAGE | polyacrylamide gel electrophoresis |
| BCA | bicinchoninic acid |
| TBST | tris buffered saline tween-20 |
| 2-DG | 2-Dexoy-D-Glucose |
| FCCP | Carbonyl cyanide 4-(trifluoromethoxy)phenylhydrazone |
| DEG | differentially expressed gene |
| GO | gene ontology |
| KEGG | Kyoto Encyclopedia of Genes and Genomes |
| FDA | Food and Drug Administration |
| GAP43 | Growth associated protein-43 |
| NF200 | Neurofilament heavy polypeptide |
| GFAP | Glial fibrillary acidic protein |
| MBP | Myelin Basic protein |
| HP | Heparin-Poloxamer Conjugates |
| EDC | 1-Ethyl-3-(3-dimethylaminopropyl) carbodiimide |
| NHS | N-Hydroxylsuccinimide |
| MATP | mono amine-terminated Poloxamer |
| TEM | Transmission electron microscopy |
| PFA | paraformaldehyde |
| GPCR | G-protein coupled receptor |
| ZO-1 | Zonula occludens-1 |
| WB | Western blot |
| P | Poloxamer |
| ECAR | extracellular acidification rate |
| OCR | oxygen consumption rate |
| PEPCK | phosphoenolpyruvate carboxykinase |
| TCA | tricarboxylic acid |
| P120 | delta 1 Catenin |
